# Insights into the structural dynamics of the secretin family (class B1) G protein-coupled receptors

**DOI:** 10.1016/j.jbc.2025.110466

**Published:** 2025-07-09

**Authors:** Ting Liu, Narisa Ria Naidoo, Eugene Agyemang, Rajan Lamichhane

**Affiliations:** 1 Department of Biochemistry & Cellular and Molecular Biology, University of Tennessee, Knoxville, Tennessee, USA; 2 Department of Pathophysiology, School of Basic Medical Sciences, Xuzhou Medical University, Xuzhou, Jiangsu, China; 3 UT-ORII Genome Science and Technology Graduate Program, University of Tennessee, Knoxville, Tennessee, USA

**Keywords:** G protein-coupled receptors, conformational dynamics, structural dynamics, single-molecule, membrane proteins

## Abstract

Class B1 G protein-coupled receptors (GPCRs) represent a significant subgroup of the GPCR family, which play key roles in cellular signaling. These receptors regulate diverse physiological processes, including metabolism, immune responses, and neuroendocrine signaling. Notably, class B1 GPCRs bind peptide hormones and are implicated in various diseases, from metabolic disorders such as diabetes to inflammatory conditions and certain cancers. Understanding the structural and functional dynamics of class B1 GPCRs is essential for the development of targeted therapies and drug design, making them a focus of extensive biomedical research. In this review, we summarize the current information regarding the dynamic structures and functions of class B1 GPCRs, elucidating their crucial roles in various physiological processes and the molecular mechanisms underlying their interactions with ligands. We explore the architectural details of the N-terminal extracellular domain (ECD) and 7-transmembrane domain (TMD), focusing on how these structural elements contribute to the receptor's conformational flexibility. This structural plasticity serves as a target for designing novel therapeutics, allowing for more precise and effective treatment of metabolic, cardiovascular, and neuroendocrine disorders.

G protein-coupled receptors (GPCRs) represent one of the largest and most diverse families of membrane proteins in eukaryotes, playing a pivotal role in cellular signaling by transducing extracellular signals into intracellular responses. These receptors are integral to a wide range of physiological processes, including vision, olfaction, immune responses, and hormone regulation, making them critical targets for approximately 36% of modern pharmaceuticals (1). GPCRs are characterized by a conserved seven-transmembrane (7TM) α-helical structure, which facilitates ligand binding and signal transduction through interactions with heterotrimeric G proteins, arrestins, and other signaling molecules. Based on sequence homology, structural features, and ligand-binding properties, GPCRs are classified into six major classes: class A (rhodopsin-like receptors), class B (secretin family receptors), class C (glutamate receptors), class D (fungal pheromone receptors), class E (cAMP receptors), and class F (frizzled/smoothened receptors) (2). Class A GPCRs represent the largest group of GPCRs in humans, comprising approximately 700 members and accounting for about 80% of all GPCRs (3), while Class B GPCRs are a small subfamily characterized by an extracellular hormone-binding domain that interacts with large peptide ligands (4).

Class B1 GPCRs, also known as the secretin-like receptor family, are a distinct subgroup within the large and diverse GPCR superfamily, comprising 15 members that play critical roles in a wide range of physiological processes. These receptors regulate numerous critical functions, such as metabolic processes, stress responses, and hormone secretion, underscoring their importance in maintaining homeostasis within the body. Class B1 GPCRs are characterized by their ability to bind peptide hormones, such as glucagon, vasoactive intestinal peptide (VIP), and glucagon-like peptide-1 (GLP-1). These GPCRs feature a large extracellular N-terminal domain crucial for ligand recognition and binding (Fig. 1). Unlike class A GPCRs, which rely primarily on ligand interactions within their transmembrane domain (TMD), class B1 receptors use a two-domain model in which the ECD first captures ligands and then positions them to engage TMD activation (5, 6, 7, 8). Figure 1 illustrates the structural differences between class B1 and class A GPCRs, highlighting key conserved domains and motifs for each class.

**Figure 1:**
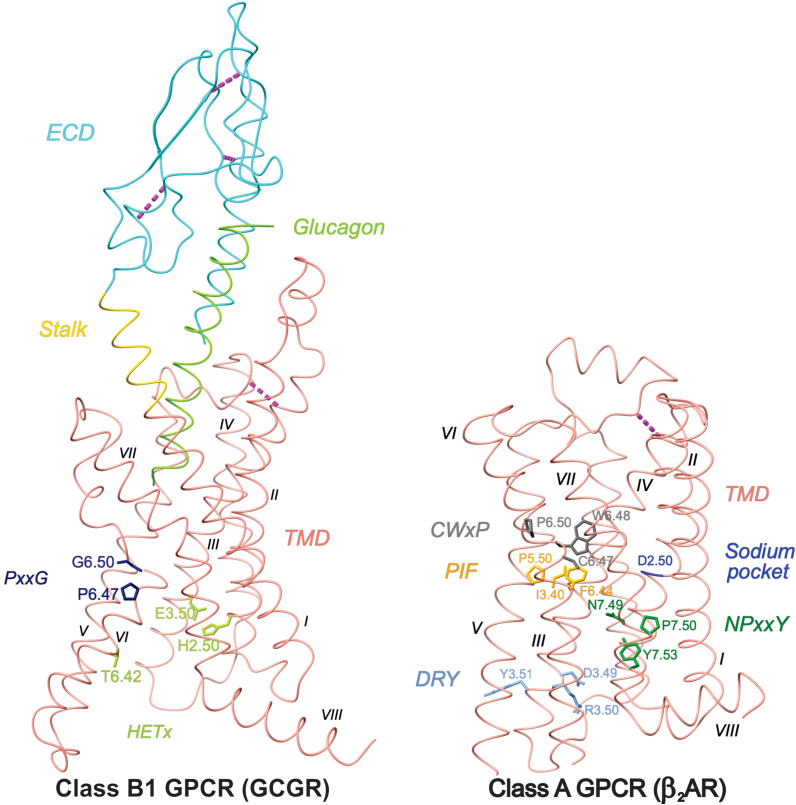
**Structural comparison of class B1 (GCGR; PDB****6LMK****) and class A (β_2_AR; PDB****2RH1****) GPCRs.** On the *left*, the class B1 receptor (GCGR) highlights conserved features including the extracellular domain (ECD), the PxxG and HETx motifs in the transmembrane helices, and multiple disulfide bonds (*magenta dashed lines*) that stabilize the ECD architecture. On the *right*, the class A receptor (β_2_AR) exhibits canonical structural motifs such as the sodium-binding site (D2.50), DRY motif (at TM, D3.49-R-3.50-Y3.51), NPxxY motif (at TM7, N7.4-P7.50-x-x-Y7.53), the PIF motif (P5.50-I3.40-F6.44), the toggle switch residue W6.48, and a flexible pivot point for outward movement of TM6 during receptor activation. Key residues are annotated using the Ballesteros–Weinstein numbering system. The figure was generated using UCSF ChimeraX.

Class B1 GPCRs are integral to energy metabolism, glucose regulation, and insulin secretion (9, 10). Recent trends indicate a growing number of clinical trials investigating GPCR modulators for metabolic diseases, oncology, and immunology, underscoring the therapeutic potential of class B1 GPCRs (1). Due to their involvement in a magnitude of human diseases such as diabetes (11), osteoporosis (12), and cardiovascular disorders (13), class B1 GPCRs have become prime targets for pharmacological intervention (14). For instance, the GLP-1 receptor, a member of class B1 GPCRs, has been central to the development of novel antidiabetic drugs due to its role in stimulating insulin secretion in response to elevated blood glucose levels (10, 15, 16).

A deeper understanding of the structure and dynamics of class B1 GPCRs is crucial for the rational design of more effective therapeutic agents. Detailed insights into receptor structures, ligand-binding domains, and conformational dynamics under various conditions are essential for identifying new therapeutic targets and designing drugs with improved efficacy and specificity. For instance, elucidating the structural basis for receptor-ligand interactions can facilitate the development of more potent and selective agonists or antagonists (17, 18). A notable example is the cryo-EM structure of GLP-1R bound to a peptide agonist (19), which revealed the active conformation of the TMD crucial for understanding receptor activation. This structural insight informed the development of small-molecule agonists, such as Danuglipron (20, 21). It also enabled *in silico* screening and rational optimization of compounds targeting the allosteric pocket of the TMD, advancing clinical candidates for the treatment of type 2 diabetes. Moreover, the exploration of biased signaling and allosteric modulators has gained momentum in GPCR drug discovery, providing new avenues for selectively targeting class B1 GPCRs with reduced side effects (1, 22).

Furthermore, class B1 GPCRs can initiate complex signaling through two main pathways upon ligand binding. The first pathway, the G protein-dependent signaling pathway, involves the activation of intracellular signaling pathways that regulate various cellular processes through a variety of Gɑ subunits and Gβγ subunits. The second pathway, the β-arrestin pathway, involves the regulation of G protein desensitization. Together, these pathways enable class B1 GPCRs to perform a wide range of physiological functions (Fig. 2). Further understanding of these pathways will provide valuable insights for potential therapeutic interventions, as manipulating these pathways could lead to the development of novel treatments for a variety of diseases. Moreover, the dynamic nature of class B1 GPCRs, characterized by their ability to adopt multiple conformational states, has significant implications for their pharmacological modulation. Understanding these dynamics, particularly the transitions between active and inactive states, is essential for developing drugs that can precisely regulate receptor activity, maximizing therapeutic benefits while minimizing side effects. This is especially relevant for allosteric modulators, which bind to sites distinct from the orthosteric ligand-binding site and influence receptor function based on its conformational state (23).

**Figure 2:**
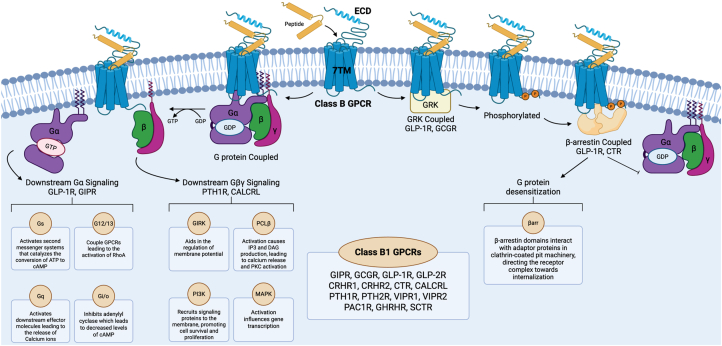
**Class B1 GPCR signaling pathways.** The two primary GPCR signaling pathways are shown: the G protein-dependent signaling pathway (*left*) and the β-arrestin pathway (*right*). In the G protein-dependent pathway, ligand binding induces a conformational change in the receptor, facilitating the exchange of GDP for GTP on the Gα subunit. This exchange leads to the dissociation of the Gα subunit from the Gβγ complex, enabling both components to activate distinct downstream signaling cascades. The Gα subunit signals through various effectors depending on its subtype, while the Gβγ subunit contributes to signaling by regulating ion channels and recruiting signaling proteins. In the β-arrestin pathway, the activated receptor recruits G protein-coupled receptor kinases (GRKs), leading to the phosphorylation of intracellular serine and threonine residues and creating a binding site for β-arrestin. The binding of β-arrestin sterically hinders further G protein coupling, leading to receptor desensitization and inhibition of G protein-mediated signaling. The Figure was created with BioRender. CALCRL: Calcitonin receptor-like receptor; CRHR1: Corticotropin-releasing hormone receptor 1; CRHR2: Corticotropin-releasing hormone receptor 2; CTR: Calcitonin receptor; ECD: Extracellular Domain; GCGR: Glucagon receptor; GHRHR: Growth hormone-releasing hormone receptor; GIPR: Gastric inhibitory polypeptide receptor; GIRK: G Protein-Activated Inwardly Rectifying Potassium Channel; GLP-1R: Glucagon-like peptide-1 receptor; GLP-2R: Glucagon-like peptide-2 receptor; GRK: G Protein-Coupled Receptor Kinase; MAPK: Mitogen-Activated Protein Kinase; PAC1R: Pituitary adenylate cyclase-activating polypeptide receptor; PTH1R: Parathyroid hormone 1 receptor; PTH2R: Parathyroid hormone 2 receptor; SCTR: Secretin receptor; TMD: Transmembrane Domain; VIPR1: Vasoactive intestinal peptide receptor 1; VIPR2: Vasoactive intestinal peptide receptor 2.

In this review, we will discuss the structures and conformational dynamics of class B1 GPCRs, as elucidated through advanced techniques such as Nuclear Magnetic Resonance (NMR), X-ray Crystallography, Cryo-Electron Microscopy (cryo-EM), and Single-molecule Förster Resonance Energy Transfer (smFRET). These techniques have significantly enhanced our comprehension of the structural intricacies and dynamic behaviors of class B1 GPCRs. This in-depth examination will provide researchers with a detailed overview of the molecular mechanisms underlying ligand recognition, receptor activation, and signal transduction in class B1 GPCRs, laying the foundation for future research directions. Furthermore, several excellent review articles have been published over time, and we recommend that readers also follow them for further insight (1, 5, 9, 15, 23, 24, 25, 26, 27, 28, 29, 30, 31, 32, 33, 34).

## Significance of understanding structure and dynamics

The structural adaptability of GPCRs enables them to recognize a wide range of extracellular ligands and interact with various intracellular partner proteins. This plasticity is a hallmark of GPCR function, and understanding this feature is crucial for elucidating their roles in downstream signaling. Biophysical techniques, such as NMR spectroscopy, have provided valuable insights into the conformational changes that occur upon ligand binding and during signal transduction (35, 36, 37, 38, 39, 40, 41). These studies are essential in elucidating the mechanisms of GPCR activation, allosteric modulation, and the specificity of receptor-partner protein interactions. A significant finding comes from NMR spectroscopy studies that reported the three-dimensional (3D) NMR structure of the ECD1 of human CRFR complexed with a high-affinity agonist, α-helical cyclic CRF (42). The study highlighted the dynamic flexibility of ECD1 loops and identified critical residues forming a hydrophobic groove that accommodates the peptide’s C-terminus. Importantly, it demonstrated how ligand binding reduces conformational entropy through loop ordering, supporting the two-domain binding model in which the ECD initially captures the peptide ligand and facilitates its engagement with the transmembrane domain as proposed previously (8).

Recent advancements in technical and instrumental methods, such as stable-isotope labeling, the development of a diverse range of NMR probes, and the use of eukaryotic expression systems have significantly enhanced our ability to investigate human receptors in their native or near-native states (35, 43). These developments introduce new avenues to explore how conformational dynamics influence GPCR signaling, including mapping allosteric communication networks and their interactions with partner proteins.

Similarly, building on the mechanistic insights for class B1 GPCRs, such as the glucagon-like peptide-1 receptor (GLP-1R) and parathyroid hormone receptor 1 (PTH1R), detailed understanding of conformational dynamics has directly informed drug discovery, offering structural principles that guide the design and optimization of next-generation therapeutics. One significant example is the cryo-EM study of GLP-1R bound to a non-peptide agonist, PF 06882961 (PDB: 6X18), which revealed a unique binding pocket within the TMD that stabilizes an active receptor conformation (44). This study demonstrated that small molecules could mimic peptide ligands by engaging specific residues to induce a conformational shift favoring G protein coupling. This finding directly informed the development of orally bioavailable GLP-1R agonists, such as Danuglipron (PF-06882961) (21). The identification of this binding mode has increased further research into non-peptide modulators for other class B1 GPCRs, expanding therapeutic options beyond traditional peptide-based drugs.

Furthermore, recent studies have shown that GPCRs exist in a dynamic equilibrium of multiple conformations, especially in their apo state, which is characterized by high conformational entropy (45). Meanwhile, the formation of a ternary complex requires a reduction in the conformational landscape, resulting in an associated entropic penalty. This intricate balance of intra- and extracellular activities underscores the entropic cost associated with GPCR signal transduction, emphasizing the complex interplay between thermodynamics, evolutionary pressures, and the structural and dynamic features that drive GPCR signaling cascades (45).

## Nuclear magnetic resonance for dynamic conformational plasticity

NMR spectroscopy was first applied to study the structure of GPCRs, including class B1 GPCRs, in the late 1990s and early 2000s (46, 47). Initially, the use of NMR in GPCR research was limited by the technical challenges associated with studying large, membrane-bound proteins. However, advancements in NMR technology and sample preparation methods have significantly improved the feasibility of investigating the structure and dynamics of GPCRs. The complexity of GPCR conformations and the early application of NMR spectroscopy to study their structure and dynamics was first stated in 2007, providing a foundation for the use of NMR in GPCR research (48, 49). Bokoch *et al.* employed NMR spectroscopy on β_2_ adrenergic receptor, a class A GPCR, to investigate the conformational changes of a salt bridge linking extracellular loops (ECLs) 2 and 3 when bound to an agonist, a neutral antagonist, and an inverse agonist (50). Using solution NMR, this study provided the first direct evidence that ligand efficacy is determined not only at the G protein interface but also on the extracellular surface of a class A GPCR. It highlighted the dynamic nature of GPCRs beyond static crystal snapshots and laid the conceptual foundation for designing drugs that target extracellular loop conformations to achieve receptor-subtype selectivity. Beyond class A receptors, NMR has also been effectively used to study class B1 receptors, particularly to characterize the dynamics of their ECDs and TMDs (Table 1).

**Table 1:** Selected class B1 GPCRs studied by NMR spectroscopy

Receptor	Ligand	Key finding	References
CRHR2	None	Antiparallel β-sheets in the ECD are stabilized by three disulfide bonds	1U34 (51)
CRHR2	Astressin	Established the two-domain model for class B1 GPCR activation	2JND4 (8)
CRHR1	α-helical cyclic CRF agonist	The hydrophobic groove formed by the ECD accommodates the C-terminal region of the agonist	2L27 (42)
GLP-1R	NNC0640	TMD cysteine labeling for ^19^F-NMR tracking dynamics	(38)
GCGR	NNC0640	TMD cysteine labeling revealing conformational shifts	(38)

**Figure 3:**
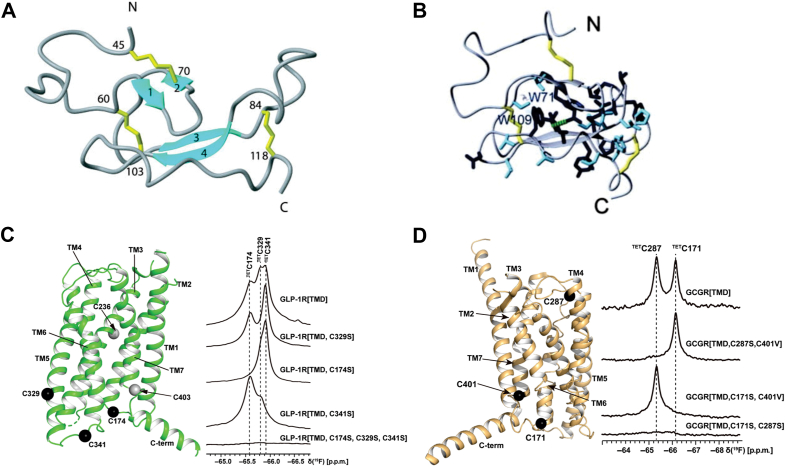
**Structural changes in Class B1 GPCRs studied using NMR spectroscopy.***A*, the lowest-energy conformer of the ECD of CRF-R2β, highlighting key secondary structural elements, including two antiparallel β-strands (cyan) and three stabilizing disulfide bonds (*yellow*), which contribute to domain stability and ligand recognition. *B*, a stereo representation of the ECD highlights conserved residues (*dark blue*) and chemically similar residues (*light blue*), with a characteristic salt bridge interaction (*green dashed line*) contributing to structural integrity. *C*, *Left*: TMD structure of GLP-1R (*green*) illustrates its seven-helix architecture with labeled cysteine residues. Cysteines modified with trifluoroethylthio (TET) for site-specific fluorescent labeling are represented as *black* spheres, while other cysteines are shown in *gray*. *Right*: One-dimensional 19F-NMR spectra of GLP-1R TMD and its cysteine-mutant variants illustrate distinct spectral shifts corresponding to changes in local chemical environments, providing insight into receptor conformational dynamics. *D*, *Left*: The crystal structure of GCGR TMD (*brown*) with TET-labeled cysteines represented as *black* spheres. *Right*: 19F-NMR spectra of GCGR TMD and its cysteine-mutant variants reveal unique spectral fingerprints correlating with structural alterations. This figure is assembled from Grace *et al.* (51) with permission from the PNAS journal (Copyright 2004, National Academy of Sciences) and Wang *et al.* (38) with permission from the FEBS journal.

Using NMR spectroscopy, the structure of the ECD1 of the mouse corticotropin-releasing factor receptor 2β (CRF-R2β), a class B1 GPCR, was determined (51). The NMR structure revealed that ECD1 consists of two antiparallel β-sheet regions, stabilized by three disulfide bonds and a salt bridge, forming a compact and stable structure (Fig. 3, *A* and *B*). This structural arrangement is crucial for the receptor’s ability to specifically bind peptide hormones. NMR chemical-shift perturbation experiments further elucidated the dynamic structural changes that occur upon ligand binding, revealing substantial shifts in specific amino acids. These shifts delineate the active role of these residues in forming the ligand-receptor interface, providing a detailed molecular understanding of ligand-induced conformational changes in CRF-R2β (51). In a related study, Huixia Wang and colleagues used in-membrane chemical modification (IMCM) for selective post-translational modification, introducing ^19^F labels at specific cysteine residues in the GLP-1R and the GCGR receptors (38) (Fig. 3, *C* and *D*). This labeling technique is crucial for NMR studies, allowing the researchers to track changes in the receptor structure upon ligand binding.

## X-ray crystallography for unraveling the structural insights of class B1 GPCRs

**Figure 4:**
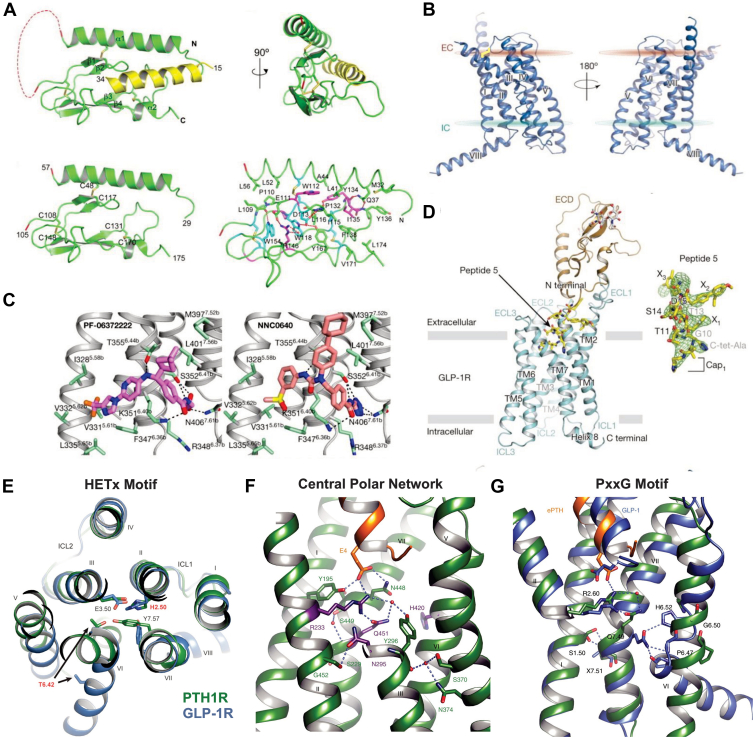
**X-ray crystallography reveals structural features in class B1 GPCRs.***A*, the extracellular domain of PTH1R in complex with PTH. A segment between residues 57 and 105 remains unresolved (*red dashed line*), and the ECD adopts a characteristic α-β-β-α fold. The first disulfide bond (C48–C117) connects the N-terminal helix to the central β-sheet layer; the second (C108–C148) links the middle and lower β-sheets, and the third (C131–C170) bridges the β-turn between strands 3 and 4 in the middle layer to the C-terminal α-helix. The general ECD fold is defined by several residues: *cyan* (invariant residues), *magenta* (residues with conservative substitutions), and *green* (non-conserved residues). The structure is stabilized by hydrogen bonds (*red dashed lines*) and a coordinated water molecule (*red sphere*). *B*, the seven-transmembrane domain of GCGR is shown in two opposing orientations, highlighting a key disulfide bond (*yellow*) linking helix III to extracellular loop 2 (ECL2). *C*, the ligand-binding pockets of GLP-1R are shown in a complex with two small-molecule ligands, PF-06372222 (*left*) and NNC0640 (*right*). The receptor is depicted in *grey*, with key interacting residues (*green*) at the binding site. Ligand atoms are color-coded: carbon (PF-06372222, *purple*; NNC0640, salmon), oxygen (*red*), nitrogen (*blue*), sulfur (*yellow*), chlorine (*green*), and fluorine (*orange*). Hydrogen bonding interactions are indicated by dashed lines. *D*, the structure of GLP-1R in complex with peptide 5 is shown, with the TMD in cyan and the ECD in *brown*. The peptide 5 agonist is depicted in stick form, with carbon in *yellow*, nitrogen in *blue*, and oxygen in *red*. The H7–Aib8 N-terminus was replaced with a "cap" (referred to as Cap1); X1 refers to α-methyl-o-fluoro-Phe substituted at the F12 position; X2 refers to 3-(4′-methoxy-2′-ethyl[1,1′-biphenyl]-4-yl)-L-alanine substituted at the V16 position; X3 refers to 5-(3,5-dimethylphenyl)-L-norvaline substituted at the S17 position; and C-tet-Ala represents C-linked tetrazolyl-Ala, which replaces E9. *E*, extracellular view of the conserved HETx motif near the cytoplasmic side of PTH1R (*green*), which stabilizes an inactive state. The active structure of GLP-1R (*blue*) is superimposed, showing a displacement of T6.42. *F*, the central polar network contains multiple ordered water molecules, highlighting conserved water-mediated interactions that play a pivotal role in the activation of class B1 GPCRs. *G*, superposition of PTH1R and the G protein-bound GLP-1R (*blue*) representing the conserved PxxG motif and residues involved in ligand recognition and receptor activation. This figure is adapted with permission from Pioszak *et al.* (52), PNAS (Copyright 2008, National Academy of Sciences), Siu *et al.* (54) (Permission from Nature Journal), Song *et al.* (55) (Permission from Nature Journal), Jazayeri *et al.* (56) (Permission from Nature Journal), and Ehrenmann *et al.* (57) (Permission from Nature Structural and Molecular Biology).

While X-ray crystallography captures molecules in a crystalline state, it has been instrumental in studying the dynamics of class B1 GPCRs by revealing their structural conformations at different stages of activation or in complexes with various ligands. By resolving structures in different functional states (inactive, partially active, and fully active conformations) when bound to different ligands, researchers can gain insight into the dynamic processes these receptors undergo during activation and signaling. X-ray crystallography has provided high-resolution snapshots of class B1 GPCR structures, revealing conformational states critical for activation (Table 2). Unlike class A GPCRs, which often lack elaborate ECDs, class B1 receptors feature a large ECD with an α-β-β-α fold. For instance, X-ray crystallography of PTH1R revealed a detailed view of its interaction with the parathyroid hormone (PTH) (52). This high-resolution structure, obtained at 1.95 Å, illustrated how PTH aligns within the receptor’s ECD, where the ECD forms a hydrophobic groove that stabilizes the amphipathic helix of the hormone, a binding mode distinct from the TMD-centric pockets of class A GPCR (52, 53). This groove emerges from a distinct three-layer α-β-β-α fold architecture of the ECD, with PTH adopting an amphipathic helical conformation that fits into the groove similar to a hot dog nestled in a bun (Fig. 4*A*). This precise structural elucidation not only delineates the hormone-receptor binding mechanism but also establishes a foundational model for understanding class B1 GPCR ligand interactions, which is essential for targeted drug design efforts.

**Table 2:** Selected class B1 GPCRs studied by X-ray crystallography

Receptor	Ligand	Key finding	References
PTH1R	PTH	ECD α-β-β-α fold with hydrophobic groove for ligand binding	3C4M (52)
PTH1R	ePTH	Critical conserved structural motifs	6FJ3 (57)
CRHR1	CRF	Non-peptide ligand binding deep within the TMD core	5K5Y (87)
GCGR	Glucagon	Inactive TMD structure with disulfide bond linking helix III to ECL2	4L6R (54)
GCGR	MK-0893	Novel extra-helical allosteric site	5EE7 (88)
GLP-1R	NAMs (PF-06372222, NNC0640)	Inactive TMD with Negative Allosteric Modulator (NAM) restricting helix VI movement	5VEX (55)
GLP-1R	Peptide agonist	Active TMD conformation with rearranged helices VI and VII	5NX2 (56)

The landmark work by Siu *et al.* presented the first crystal structure of a class B1 GPCR, specifically the glucagon receptor (GCGR), offering insights into its inactive state and the architecture of its ligand-binding domain (54). Notably, the study elucidated the structure of the GCGR TM domain at a resolution of 3.4 Å, showing a typical TM fold (Fig. 4*B*). In a separate study, the crystal structure of the Human GLP-1 receptor (GLP-1R) in complex with two negative allosteric modulators (NAMs), PF-06372222 and NNC0640, was resolved (55). These structures revealed a common binding pocket for NAMs located outside helices V–VII, near the intracellular half of the receptor, a region critical for modulating the receptor's activity. Binding of the NAM to this pocket stabilizes the receptor in an inactive conformation by limiting the movement of the intracellular tip of helix VI, a region typically associated with GPCR activation (Fig. 4*C*). Similarly, computational and mutagenesis studies suggest that positive allosteric modulators (PAMs) target a similar region within a different sub-pocket, facilitating a conformational change that enhances G protein coupling. Additionally, another study resolved the crystal structure of the full-length GLP-1R bound to a truncated peptide agonist (56). In the structure, the agonist maintains an α-helical conformation within the receptor-binding pocket, aligning with the transmembrane helices. This arrangement allows for a substantial rearrangement of the transmembrane helices, particularly TMD6 and TMD7, promoting the receptor's transition to an active state and enabling G protein coupling and signaling (Fig. 4*D*).

In another study, the X-ray structure of PTH1R in complex with an engineered PTH mimetic agonist ePTH revealed detailed molecular features of class B1 GPCRs (57). The receptor adopts a conformation and fold consistent with other class B1 GPCRs, displaying conserved activation-related structural motifs. Among them is the HETx motif, which consists of H2.50, E3.50, and T6.42. Notably, the polar lock formed between E3.50 and T6.42 is disrupted upon activation of the receptor (Fig. 4*E*) (58, 59, 60). The structure also highlights a network of ordered water molecules within the orthosteric binding pocket, which mediate and extend the central polar interaction networks essential for ligand recognition and receptor activation (Fig. 4*F*). Additionally, the conserved PxxG motif and other residues involved in ligand recognition and conformational transitions are also presented and compared through alignment with the previously solved structure of the GLP-1R-GLP1 complex (PDB 5VAI) (Fig. 4*G*). Together, these findings underscore the unique and cooperative role of ECD and TMD interactions in class B1 GPCR activation (57).

## Cryo-electron microscopy is advancing the structural understanding of class B1 GPCRs

**Figure 5:**
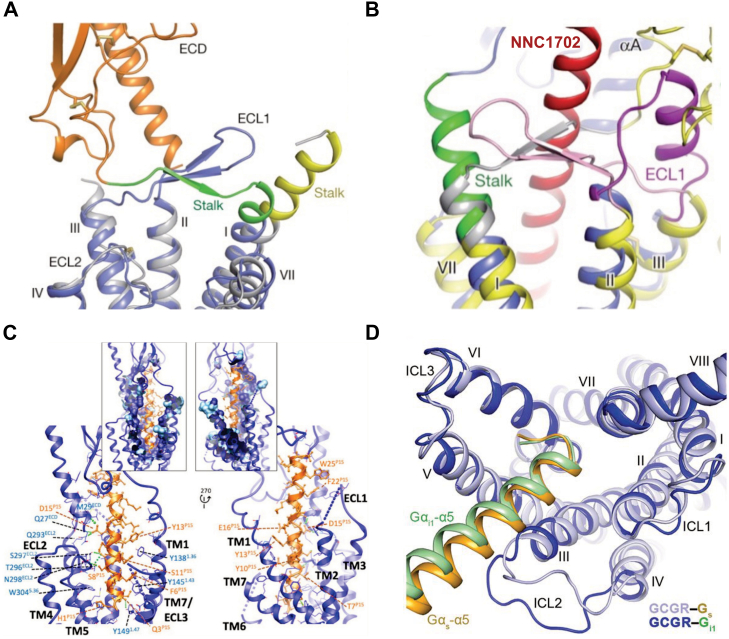
**Cryo-electron microscopy reveals the structural and conformational transitions of GCGR.***A*, a comparison of the full-length GCGR and its TMD highlights key differences in the stalk conformation. In the complete receptor model, the ECD is depicted in *orange*, the stalk in *green*, and the TMD in *blue*. In contrast, the isolated TMD structure is depicted with a *yellow* stalk and a *gray* TMD. *B*, cartoon representations of the GCGR–NNC1702 complex (*blue*) and the GCGR–NNC0640–mAb1 complex (*yellow*) are shown, with the peptide NNC1702 highlighted in *red*. In the GCGR–NNC1702 structure, the stalk is colored *green*, and the first extracellular loop (ECL1) is shown in magenta. *C*, the spatial arrangement of GCGR residues located within 5 Å of P15 is depicted, with the receptor’s backbone represented as a ribbon structure. Hydrogen bonds are denoted by *greendashed lines*. Insets display surface marking residues within 5 Å proximity. *D*, cartoon representation of the GCGR–Gs (*light blue* for GCGR, *yellow* for Gαs) and GCGR-Gi1 (*dark blue* for GCGR, *green* for Gαi1) complexes in an intracellular view, illustrating the differences in transmembrane helical bundle conformation and the positioning of the C-terminus of the Gα α5 helix. This figure is assembled from Zhang *et al.* (67, 68) with permission from *Nature Journal*, Chang *et al.* (69) with permission from the *Journal of Biological Chemistry*, and Qiao *et al.* (58), Reprinted with permission from AAAS.

Cryo-electron microscopy (cryo-EM) studies have significantly advanced our understanding of class B1 GPCRs, providing detailed insights into their structure, activation mechanisms, and interactions with ligands and G proteins. Since the first near-atomic resolution structure was solved in 2017, all 15 members of class B1 GPCR structures have been determined using cryo-EM (19, 58, 61, 62, 63, 64, 65, 66, 67, 68, 69) (Table 3). Unlike class A GPCRs, which rely on TMD rearrangements (*e.g.*, β_2_-adrenergic receptor), class B1 receptors exhibit extensive ECD-TMD coordination. A cryo-EM study of GCGR complexed with the NAM NNC0640 and inhibitory antibody mAb1 revealed the ECD of GCGR adopting an α-β-β-α fold. This fold is linked to the transmembrane domain *via* a 12-residue stalk (67). This stalk adopts a β-strand conformation, contrasting with the α-helix observed in prior GCGR TMD structures. At the same time, the first extracellular loop (ECL1) forms a β-hairpin that engages with the stalk to produce a compact β-sheet structure. This structural configuration is critical for modulating peptide ligand binding and receptor activation (Fig. 5*A*). Further research involving a GCGR complex with the glucagon analog NNC1702 revealed that the ECD and TMD of GCGR in the bound structure maintain conformations similar to the inactive GCGR state (68). However, significant shifts in the relative orientations of the ECD and TMD were observed, suggesting conformational alterations upon ligand binding. Particularly, the stalk region (residues G125–K136) transitions from an extended β-strand in the inactive state to a 3-turn α-helical structure upon NNC1702 binding. This transition is pivotal for ligand interaction and receptor activation (Fig. 5*B*). Additionally, ECL1 (residues S203–A220) shifts from a β-hairpin to an extended configuration, aligning with helices II and III. This shift promotes the dissociation of ECL1 from the stalk, further promoting ligand binding and receptor activation (68).

**Table 3:** List of class B1 GPCRs studied by cryo-EM

Receptor	Ligand	Key finding	References
GIPR	GIP	GIP forms a straight helix, with its N-terminus penetrating the receptor TMD and its C-terminus interacting with the ECD and ECL1.	7DTY (65)
GCGR	Glucagon	Identified a binding cavity in GCGR for G protein coupling	6LMK (58)
GCGR	Peptide 15	Observed increased mobility of the GCGR extracellular domain and a distinct conformation of ECL3	6WHC (69)
GCGR	Partial agonist, NNC1702	The stalk region and the first extracellular loop undergo major conformational changes	5YQZ (68)
GLP-1R	GLP-1	A sharp kink in the middle of transmembrane helix 6	5VAI (19)
GLP-2R	GLP-2	The middle region of GLP-2 engages with TM1 and TM7 more extensively than with ECL2	7D68 (89)
CRFR	UCN1	UCN1 adopts a single straight helix with its N terminus dipped into the receptor transmembrane bundle	6PB0, 6PB1 (64)
CTR	Peptide agonist	A large outward movement of transmembrane helix 6 and 7	5UG7 (61)
CTR	Calcitonin	The extracellular loops (ECLs) 2 and 3 of CTR has been resolved	6NIY (90)
CTR (AMYR)	Amylin or Calcitonin	The two peptide hormones activate AMYRs by distinct mechanisms	7TYF, 7TYX, 7TZF (66)
CGRPR	CGRP/RAMP1	The extracellular loop 2 of CLR is stabilizes by the receptor activity-modifying protein transmembrane domain	6E3Y (62)
CALCRL (AMR)	Adrenomedullin1, Adrenomedullin2	The orientation and mobility of the ECDs and the position of ECL3 vary depending on the receptor	6UUN, 6UUS, 6UVA (91)
PTH1R	PTH	A partial unwinding of the carboxyl terminus and a sharp kink of transmembrane helix 6	6NBF (63)
PTH2R	TIP39	The N terminus of TIP39 plays an important role in PTH2R activation	7F16 (92)
VIP1R	PACAP27	PACAP27 engages VIP1R with its N-terminus inserting into the ligand binding pocket at the transmembrane bundle of the receptor	6VN7 (93)
VIP2R	PACAP27	The N-terminal α-helix of VIP2R adopts a unique conformation that deeply inserts into a cleft between PACAP27 and the extracellular loop 1, thereby stabilizing the peptide-receptor interface.	7VQX (94)
SCTR	Secretin	The ECD of SerR exhibited a unique organization relative to TM core	6WZG (95)
GHRHR	GHRH	The α-helical GHRH forms an extensive and continuous network of interactions with GHRHR	7CZ5 (96)
PAC1R	Maxadilan/PACAP38	The ECD accommodates ligands in different orientations while ECL1 protrudes to further anchor the ligand bound in the orthosteric site	6M1H, 6M1I, 6P9Y (97, 98)

Using cryo-EM, researchers also found that the dual agonist, P15, a modified glucagon (GCG) peptide, interacts differently with GCGR compared to the standard GCG agonist. This interaction, particularly involving regions like the ECL1 and the extracellular half of transmembrane domain 1, induces a distinct conformation that may enhance receptor activation and influence its pharmacological profile (Fig. 5*C*) (69). GCGR primarily interacts with G proteins to transmit signals from glucagon to intracellular pathways. GCGR’s ability to interact with various G proteins underscores the complexity of glucagon signaling and highlights potential targets for therapeutic interventions. Anna Qiao and colleagues obtained high-resolution structures of GCGR in complex with glucagon bound to two different G proteins, Gs and Gi1. These structures revealed that both GCGR-Gs and GCGR-Gi1 complexes exhibit similar conformational changes required for receptor activation and G protein coupling. However, notable differences were observed in how the receptor interacts with Gs and Gi, particularly in the intracellular loops and the C-terminal regions of the G proteins (Fig. 5*D*) (58). These findings provide critical insights into GCGR’s structural dynamics upon ligand and G protein engagement, revealing specific molecular interfaces that can be precisely targeted to develop new therapeutics capable of fine-tuning glucagon signaling for improved metabolic and cardiovascular outcomes.

## Single-molecule fluorescence reveals real-time conformational dynamics at the molecular level

Single-molecule fluorescence has become a promising tool for studying the conformational dynamics of GPCRs, enabling researchers to observe real-time structural changes at the molecular level (41, 70, 71, 72, 73, 74, 75, 76, 77). Single-molecule spectroscopy was first applied to GPCRs in 2001, revealing conformational substates and basal activity of the β_2_-adrenergic receptor in solution (77). Single-molecule Förster Resonance Energy Transfer (smFRET) has since been employed to uncover the dynamic behavior of various GPCR regions, including their transmembrane helices (70, 76, 78, 79), extracellular domains (80, 81), and C-terminal domains (82, 83). These studies established smFRET as a powerful approach for capturing real-time conformational changes at the single-molecule level, overcoming the averaging limitations of ensemble-based techniques. Additionally, this technique has been employed to explore the kinetics of GPCR dimerization (84, 85) and ligand binding (86). Extending this technique to class B1 GPCRs required adaptations to account for their larger ECDs and complex peptide-binding mechanisms, which contrast with the compact orthosteric pockets characteristic of class A receptors.

**Figure 6:**
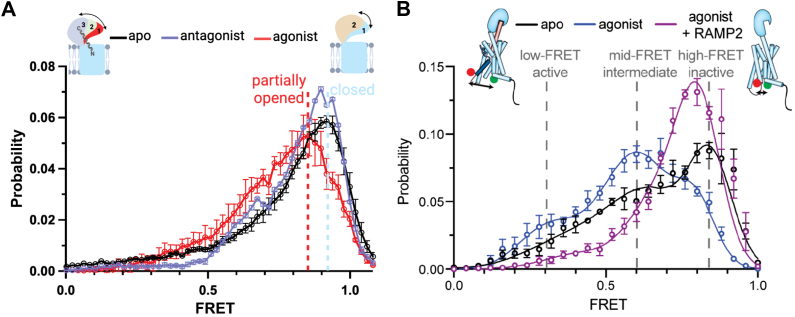
**Conformational dynamics of GCGR revealed by smFRET.***A*, smFRET population histograms showing GCGR’s ECD dynamics under different ligand conditions: Apo (*black*), glucagon-bound (*red*), and MK0893-bound (*green*). *Dashed vertical lines* represent the highest FRET states observed for each complex. The cartoon depicts the ECD positions (1, 2, 3) based on the populated FRET states, with solid double-headed *arrows* indicating movement between high and low FRET states. For apo and antagonist MK0893-bound GCGR (*upper right*), the ECD transitions between closed (1, *light blue*) and open (2, *light orange*) conformations, with a preference for the closed conformation. Binding of glucagon’s C-terminus to the ECD (*upper left*) triggers a partial opening of the ECD (1–3, *red* to *light purple*), which allows glucagon’s N terminus to interact with the orthostatic pocket. The *gray* lipid bilayer represents the cell membrane. *B*, the cartoon illustrates the conformational dynamics of GCGR’s TMD6 (*light blue*). smFRET experiments were performed using site-specifically labeled donor (LD555, *green sphere*) and acceptor (LD655, *red sphere*) fluorophores at positions 265C (TM4) and 345C (TM6). smFRET experiments reveal that in the absence of an orthosteric agonist, the intracellular ends of TM4 and TM6 predominantly exhibits a high-FRET state (∼0.83, *black*). Upon binding the agonist peptide ZP3780, this high-FRET state shifts to a dominant mid-FRET state (*blue*). The presence of RAMP2 significantly reduces the mid-FRET intermediate and low-FRET active conformations induced by the agonist peptide, shifting the distribution toward an inactive-like conformation (*purple*). The figure is assembled from Liu *et al.* (80) with permission from the Journal of Biological Chemistry and Krishna Kumar *et al.* (71) with permission from Cell.

For class B1 GPCRs, two leading models propose that peptide ligands initiate receptor activation either through binding to the extracellular domain or by directly engaging the transmembrane domain (6, 9). smFRET studies have demonstrated that both the ECD and TMD of class B1 GPCRs undergo conformational changes upon ligand binding (Table 4). Furthermore, our recent study supports a cooperative mechanism where the ECD initially captures the peptide ligand (glucagon), facilitating its subsequent engagement with the TMD binding pocket, rather than exclusively binding to either domain. Specifically, we observed that the GCGR ECD dynamically transitions between closed and a partially open state in the absence of glucagon (Fig. 6*A*) (80). Upon glucagon binding, the ECD adopts a fully open conformation and displays dynamic behavior, increased conformational flexibility compared to apo-GCGR. This previously unreported dynamic behavior suggests a mechanism for fine-tuning ligand recognition and receptor activation, contributing significantly to our understanding of GCGR’s ligand binding dynamics and the crucial role of its ECD.

**Table 4:** List of class B1 GPCRs studied by smFRET

Receptor	Ligand	Key finding	References
GCGR	ZP3780/RAMP2	TMD6 shifts to active-like and active states	8FU6 (71)
GCGR	Glucagon/MK0893	ECD transitions from closed to open states	(80)

In addition to the ECD, the TMDs also undergo conformational changes during GCGR activation. As reported in a recent study (71), the apo state of GCGR displays a wide distribution of FRET efficiencies, with a major FRET peak centered around ∼0.83, indicative of an inactive state (Fig. 6*B*, black). Upon binding of the full agonist peptide ZP3780, the distribution of FRET values remains heterogeneous, but shifts toward a dominant mid-FRET peak at ∼0.63. At the same time, the inactive high-FRET state population decreases while the active low-FRET (∼0.32) population increases, indicating GCGR activation (Fig. 6*B*, dark blue). When RAMP2, a receptor activity-modifying protein that acts as a NAM of GCGR, is added to the agonist-bound receptor, it suppresses the fully active and agonist-associated intermediate states of TM6, shifting the conformational equilibrium towards an inactive-like conformation (Fig. 6*B*, purple). These smFRET experiments have provided significant insights into the dynamic conformational changes of the glucagon receptor, shedding light on its activation mechanism. By capturing real-time structural transitions, smFRET reveals details on the influence of ligand binding on GCGR’s structural dynamics, which is essential for understanding the molecular basis of class B1 GPCR function. This knowledge is crucial for developing targeted therapeutic strategies for metabolic disorders such as diabetes, enabling the rational design of ligands that modulate receptor activity with high precision.

## Conclusions

Class B1 GPCRs exhibit unique structural dynamics, with coordinated interactions between the ECD and TMD, enabling peptide ligand recognition and receptor activation. Current biophysical studies support a cooperative binding model, in which ECD flexibility facilitates ligand engagement with the TMD, distinct from the TMD-centric mechanisms seen in class A GPCRs. Structural insights into receptor conformation and dynamics provide a strong foundation for the development of new drugs and the design of more precise therapeutics. Current research primarily focuses on *in vitro* studies and non-native environments such as detergent micelles. What remains largely unexplored is how these receptors behave in their native membrane context, and more importantly, in the complex environment of living cells. In this regard, future research should focus on leveraging the advanced tools mentioned in this review. This includes applying time-resolved cryo-EM to capture transient intermediate states, utilizing enhanced smFRET with faster camera sensors for real-time monitoring of conformational dynamics within native membranes, and developing computational models capable of predicting biased signaling pathways. Additionally, integrating these techniques within physiologically relevant cellular contexts and developing new biosensors or high-throughput screening methods to probe receptor–effector interactions will be crucial for fully elucidating class B1 GPCR functions and unlocking their therapeutic potential.

## Conflict of interest

The authors declare no conflicts of interest with the contents of this article.
